# The Continuity of the Meter: The Redefinition of the Meter and the Speed Of Visible Light

**DOI:** 10.6028/jres.092.001

**Published:** 1987-02-01

**Authors:** D. A. Jennings, R. E. Drullinger, K. M. Evenson, C. R. Pollock, J. S. Wells

**Affiliations:** National Bureau of Standards Boulder, CO 80303

**Keywords:** frequency, laser, meter, speed of light

## Abstract

The product of the frequency and wavelength of the *i*^th^ hyperfine component of the 11-5, R(127) transition of ^127^I_2_ yields a value for the speed of visible red light. This value of *c*, the most accurate ever measured for visible light, agrees with the value defined in the redefinition of the meter within the 3*σ* error limits of the krypton length standard.

The speed of light has intrigued scientists for several centuries and during the short quarter century of the laser it has not been different. The measurement of the speed of light, *c*, by a group of scientists at the National Bureau of Standards in 1972 [1]^1^ reported a value for c from the product of the wavelength and frequency of a 3.39 μm He-Ne laser which was limited in accuracy by the uncertainty in the krypton length standard. This definitive measurement for *c* followed by other confirmative measurements prompted the international scientific community, through the Comité Consultatif pour la Definition du Metre (CCDM), to a new definition for the meter. The new definition for the meter, adopted by the Conférence Générale des Poids et Mesures (CGPM) in October 1983 reads, “The meter is the length of the path travelled by light in vaccum during a time interval of 1/299 792 458 of a second.” [2] This definition for the meter fixes the speed of light to be *exactly* 299 792 458 m/s. With this definition the meter could be realized from the wavelength of any coherent optical source whose frequency is known, for example, a laser which is stabilized to a narrow atomic or molecular absorption for which the frequency is known. The wavelength λ would be determined from the relation λ*=c*/*v*, where *c* is the fixed value of the speed of light, and *v* is the measured frequency of the transition. Since the measurement in 1972 there have been four speed of light measurements [3–6]; two at a wavelength of 3.39 μm and two at a wavelength of 9.31 μm. These measurements have been summarized [7], and the average value for the speed of light is 299 792 458.1 m/s with a fractional uncertainty of ±4×10^−9^ (3*σ*), which is the recognized uncertainty in the realization of the meter from the krypton definition.

We are reporting a value of *c* using visible rather than infrared radiation. This has been made possible by the absolute frequency measurement of the visible He-Ne laser stabilized on the *i*^th^ hyperfine component of the 11-5, R(127) transition of the molecule ^127^I_2_ [8]. The reported value for the frequency of this transition is 473 612 214.830 MHz with a fractional uncertainty of 1.6×10^−10^. The wavelength for this transition is obtained from four published values which are 632 991 399.0±0.8 fm [9], 632 991 399.8±0.9 fm [10], 632 991 400.0±1.2 fm [11], and 632 993 398.0±3 fm [12]. These four wavelengths are from direct wavelength measurements referred to the wavelength of krypton. The weighted average of these measurements is 632 991 399.4±0.6 fm. The value for the speed of light is, of course, the product of the frequency and wavelength and is *c*= 299 792 458.6±0.3 m/s, with a one sigma uncertainty.

This value of *c*, the most accurate ever measured for visible light, is in good agreement with the defined value of *c* proposed by the CCDM within the recognized uncertainties in the use of the krypton length standard (±1.2 m/s, 3*σ*) [11], and was the final confirmation in the choice of the new definition for the standard of length.

The fractional uncertainty of the meter realized through the new definition and use of a laser stabilized on either this frequency measured iodine transition [8], or another in the yellow region [13] is 10 times smaller than the uncertainty as realized through the krypton definition and would represent a tenfold improvement in accuracy for length metrology. Future frequency measurements in the visible will undoubtedly be even more accurate, ultimately being limited by the time standard itself. In fact, length metrology need not be limited by the frequency measurement of the laser used to realize the meter. Thus, with the new definition, a new era of length metrology is at hand, one in which the uncertainty will not be due to the length standard but with the measurement techniques.

It is always interesting to speculate on the dispersion of the speed of light. The most accurate values to date are at the CH_4_ transition at 88.4 THz and the one reported here. Using formula

$$\frac{\Delta c}{\Delta v}=\frac{c_{88}-c_{473}}{v_{88}-v_{473}}$$

and using the best values (CGPM for 88 THz and this paper for 473 THz) gives the result

$$\Delta c/\Delta v<1.5\times 10^{-15}m/s^{2}.$$

## Footnotes

1 Figures in brackets indicate literature references.

## Appendices

### Appendix I. Practical Realization of the Definition of the Meter

The following recommendations for the practical realization of the definition of the meter were adopted by the International Committee for Weights and Measures (Comité International de Poids et Mesures, CIPM) in 1983.

The CIPM *recommends* that the meter be realized by one of the following methods:

- by means of the length *l* of the path travelled in vacuum by a plane electromagnetic wave in a time *t;* this length is obtained from the measured time *t*, using the relation *l =c* · *t* and the value of the speed of light in vacuum *c* = 299 792 458 m/s;
- by means of the wavelength in vacuum λ of a plane electromagnetic wave of frequency *f*; this wavelength is obtained from the measured frequency *f*, using the relation *λ = c/f*· and the value of the speed of light in vacuum *c* =299 792 458 m/s;
- by means of one of the radiations from the list below, whose stated wavelength in vacuum or whose stated frequency can be used with the uncertainty shown, provided that the given specifications and accepted good practice are followed;

and that in all cases any necessary corrections be applied to take account of actual conditions such as diffraction, gravitation, or imperfection in the vacuum.

#### List of recommended radiations, 1983

In this list, the values of the frequency *f* and of the wavelength λ should be related exactly by the relation λ*f*=*c*, with *c* =299 792 458 m/s but the values of λ are rounded.

##### 1. Radiations of Lasers Stabilized by Saturated Absorption^a^

###### 1.1. Absorbing molecule CH_4_, transition *v*_3_, P(7), component $F_{2}^{\left(2\right)}$

The values

*f* = 88 376 181 608 kHz
λ = 3 392 231 397.0 fm

with an estimated overall relative uncertainty of ± 1.3×10^−10^ [which results from an estimated relative standard deviation of 0.44×10^−10^] apply to the radiation of a He-Ne laser stabilized with a cell of methane, within or external to the laser, subject to the conditions:

methane pressure ⩽ 3 Pa
mean one-way axial intracavity surface power density^b^ ⩽ 10^4^ W · m^−2^
radius of wavefront curvature ⩾ 1 m
inequality of power between counter-propagating waves ⩽ 5%.

###### 1.2. Absorbing molecule ^127^I_2_, transition 17-1, P(62), component o

The values

*f* = 520 206 808.51 MHz
λ = 576 294 760.27 fm

with an estimated^c^ overall relative uncertainty of ±6×10^−10^ [which results from an estimated relative standard deviation of 2×10^−10^] apply to the radiation of a dye laser (or frequency-doubled He-Ne laser) stabilized with a cell of iodine, within or external to the laser, having a cold-finger temperature of 6 °C±2 °C.

###### 1.3. Absorbing molecule ^127^I_2_, transition 11-5, R(127), component i

The values

*f*=473 612 214.8 MHz
λ = 632 991 398.1 fm

with an estimated overall relative uncertainty of ± 1×10^−9^ [which results from an estimated relative standard deviation of 3.4×10^−10^] apply to the radiation of a stabilized He-Ne laser containing an iodine cell, subject to the conditions:

cell-wall temperature between 16 °C and 50 °C with a cold-finger temperature of 15 °C±1 °C
one-way intracavity beam power^b^ 15 mW±10 mW
frequency modulation amplitude, peak to peak, 6 MHz±1 MHz.

###### 1.4. Absorbing molecule ^127^I_2_, transition 9-2, R(47), component o

The values

*f* = 489 880 355.1 MHz
λ = 611 970 769.8 fm

with an estimated overall relative uncertainty of ±1.1×10^−9^ [which results from an estimated relative standard deviation of 3.7×10^−10^] apply to the radiation of a He-Ne laser stabilized with a cell of iodine, within or external to the laser, having a cold-finger temperature of −5 °C±2 °C.

###### 1.5. Absorbing molecule ^127^I_2_, transition 43-0, P(13), component a_3_ (sometimes called component s)

The values

*f* = 582 490 603.6 MHz
λ = 514 673 466.2 fm

with an estimated overall relative uncertainty of ±1.3×10^−9^ [which results from an estimated relative standard deviation of 4.3×10^−10^] apply to the radiation of an Ar^+^ laser stabilized with a cell of iodine, within or external to the laser, having a cold-finger temperature of −5 °C±2 °C.

### Appendix II. Decisions of the Conférence Générale des Poids et Mesures (CGPM) Regarding the Definition of the Meter

#### 1st CGPM, 1889

“*Sanction of the international prototype of the meter*.

“The General Conference, considering

“..that … the fundamental measurements of the international and national prototypes of the meter [has] been made with all the accuracy and reliability that the present state of science permits; that the international and national prototypes of the meter are made of an alloy of platinum with 10 percent iridium, to within 0.0001; the equality in length of the international Meter … with the length of the Meter kept in the Archives of France;

“that the differences between the national Meters and the international Meter lie within 0.01 millimeter and that these differences are based on a hydrogen thermometer scale which can always be reproduced..;

“that the international Meter … and the national Meters … fulfill the requirements of the Meter Convention,

“**Sanctions** The Prototype of the meter chosen by the Comité International des Poids et Mesures (CIPM). This Prototype, at the temperature of melting ice, shall henceforth represent the metric unit of length.”

#### 7th CGPM, 1927

“Definition of the meter by the international Prototype.

The unit of length is the meter, defined by the distance, at 0°, between the axes of the two central lines marked on the bar of platinum-iridium kept at the BIPM, and declared Prototype of the meter by the 1st CGPM, this bar being subject to standard atmospheric pressure and supported on two cylinders of at least one centimeter diameter, symmetrically placed in the same horizontal plane at a distance of 571 mm from each other.”

#### 11th CGPM, 1960

“Considering that the international Prototype does not define the meter with an accuracy adequate for the present needs of metrology, [the CGPM] decides that it is moreover desirable to adopt a natural and indestructible standard.

“The meter is the length equal to 1 650 763.73 wavelengths in vacuum of the radiation corresponding to the transition between the levels 2 p_10_ and 5 d_5_ of the krypton 86 atom.”

#### 15th CGPM, 1975

*considering* the excellent agreement among the results of wavelength measurements on the radiations of lasers locked on a molecular absorption line in the visible or infrared region, with an uncertainty estimated at ±4×10^−9^ which corresponds to the uncertainty of the realization of the meter,

*considering* also the concordant measurements of the frequencies of several of these radiations,

*recommends* the use of the resulting value for the speed of propagation of electromagnetic waves in vacuum *c* = 299 792 458 meters per second.

#### 17th CGPM, 1983

“Considering: that the present definition does not allow a sufficiently precise realization of the meter for all requirements,

that progress made in the stabilization of lasers allows radiations to be obtained that are more reproducible and easier to use than the standard radiation emitted by a krypton 86 lamp,

that progress made in the measurement of the frequency and wavelength of these radiations has resulted in concordant determinations of the speed of light whose accuracy is limited principally by the realization of the present definition of the meter,

that wavelengths determined from frequency measurements and a given value for the speed of light have a reproducibility superior to that which can be obtained by comparison with the wavelength of the standard radiation of krypton 86,

that there is an advantage, notably for astronomy and geodesy, in maintaining unchanged the value of the speed of light recommended in 1975 by the 15th CGPM in its Resolution 2 (*c* =299 792 458 m/s),

that a new definition of the meter has been envisaged in various forms all of which have the effect of giving the speed of light an exact value, equal to the recommended value, and that this introduces no appreciable discontinuity into the unit of length, taking into account the uncertainty of ±4×10^−9^ of the best realizations of the present definition of the meter,

that these various forms, making reference either to the path travelled by light in a specified time interval or to the wavelength of a radiation of measured or specified frequency, have been the object of consultations and deep discussions, have been recognized as being equivalent and that a consensus has emerged in favor of the first form,

that the CCDM is now in a position to give instructions for the practical realization of such a definition, instructions which could include the use of the orange radiation of krypton 86 used as standard up to now, and which may in due course be extended or revised,

“The 17th CGPM invites the CIPM to draw up instructions for the practical realization of the new definition of the meter to choose radiations which can be recommended as standards of wavelength for the interferometric measurement of length and to draw up instructions for their use.”

[See “Practical Realization of the Definition of the Meter,” *above*.]

*Appendices 1 and 2 are taken from NBS Special Publication 330, The International System of Units (SI) and are the official translations from the minutes of the General Conferences on Weights and Measures*.
